# Rosiglitazone and a β_3_-Adrenoceptor Agonist Are Both Required for Functional Browning of White Adipocytes in Culture

**DOI:** 10.3389/fendo.2018.00249

**Published:** 2018-05-30

**Authors:** Jon Merlin, Masaaki Sato, Ling Yeong Chia, Richard Fahey, Mohsen Pakzad, Cameron J. Nowell, Roger J. Summers, Tore Bengtsson, Bronwyn A. Evans, Dana S. Hutchinson

**Affiliations:** ^1^Drug Discovery Biology, Monash Institute of Pharmaceutical Sciences, Monash University, Parkville, VIC, Australia; ^2^Department of Toxicology and Pharmacology, Faculty of Pharmacy, Pharmaceutical Sciences Research Center, Tehran University of Medical Sciences, Tehran, Iran; ^3^Department of Molecular Biosciences, The Wenner-Gren Institute, Stockholm University, Stockholm, Sweden; ^4^Department of Pharmacology, Monash University, Clayton, VIC, Australia

**Keywords:** adipocyte, beta adrenergic receptors, uncoupling protein 1, adrenoceptor, seahorse xf96 analysis, rosiglitazone, CL316243

## Abstract

The recruitment of brite (or beige) adipocytes has been advocated as a means to combat obesity, due to their ability to phenotypically resemble brown adipocytes (BA). Lineage studies indicate that brite adipocytes are formed by differentiation of precursor cells or by direct conversion of existing white adipocytes, depending on the adipose depot examined. We have systematically compared the gene expression profile and a functional output (oxygen consumption) in mouse adipocytes cultured from two contrasting depots, namely interscapular brown adipose tissue, and inguinal white adipose tissue (iWAT), following treatment with a known browning agent, the peroxisome proliferator-activated receptor (PPARγ) activator rosiglitazone. Prototypical BA readily express uncoupling protein (UCP)1, and upstream regulators including the β_3_-adrenoceptor and transcription factors involved in energy homeostasis. Adipocytes from inguinal WAT display maximal UCP1 expression and mitochondrial uncoupling only when treated with a combination of the PPARγ activator rosiglitazone and a β_3_-adrenoceptor agonist. In conclusion, brite adipocytes are fully activated only when a browning agent (rosiglitazone) and a thermogenic agent (β_3_-adrenoceptor agonist) are added in combination. The presence of rosiglitazone throughout the 7-day culture period partially masks the effects of β_3_-adrenoceptor signaling in inguinal white adipocyte cultures, whereas including rosiglitazone only for the first 3 days promotes robust β_3_-adrenoceptor expression and provides an improved window for detection of β_3_-adrenoceptor responses.

## Introduction

The remarkable prevalence of obesity worldwide has sparked considerable interest in therapeutic strategies that are effective and safe in promoting weight loss. In conjunction with lifestyle modification, such strategies range from surgical intervention to reduce stomach capacity, through to pharmaceutical interventions aimed primarily at modulating neural pathways that affect food/caloric intake. There are no pharmacological interventions that primarily act on adipose tissue, despite its obvious role in obesity. There are two types of adipose tissue with distinct functions: white adipose tissue (WAT) that stores chemical energy in the form of triacylglycerol, and brown adipose tissue (BAT) that releases chemical energy in the form of heat (thermogenesis).

Classical BAT depots have been studied extensively in adult rodents. They are highly innervated and are activated by centers in the brain responsive to cold exposure, leading to the release of norepinephrine (NE) from sympathetic nerves. Upon binding of NE to BAT β_3_-adrenoceptors (β_3_-ARs), increased levels of intracellular cyclic AMP (cAMP) promote lipolysis, and this breakdown of triglycerides leads to release of free fatty acids that upregulate and activate uncoupling protein 1 (UCP1). Activated UCP1 uncouples mitochondrial respiration leading to heat generation, thus β_3_-AR signaling increases respiration and non-shivering thermogenesis, with prototypical BAT adipocytes being remarkably rich in mitochondria (1).

β_3_-AR signaling in response to NE or synthetic receptor agonists occurs almost exclusively in peripheral tissues, including adipose tissue/adipocytes, bladder, and gastrointestinal tract. While there is detectable expression in the rodent brain (2), and central administration of β_3_-AR agonists directly into hypothalamic/third ventricle regions can reduce food intake and weight (3, 4) and increase c-fos immunoreactivity (5), it is unlikely that peripheral administration of β_3_-AR agonists such as CL316243 and mirabegron would have direct central effects, since they do not readily cross the blood–brain barrier (6, 7). Any effects that peripheral administration of β_3_-AR agonists has centrally are thought to occur indirectly through fatty acids liberated from lipolysis in peripheral adipose tissues.

In addition to prototypical white or brown adipocytes (BA), beige or brite adipocytes have been described (8, 9). These cells reside in WAT depots but can be “browned” by various stimuli, most notably cold exposure or activation of β-AR signaling, and by the peroxisome proliferator-activated receptor (PPARγ) agonist rosiglitazone. Activation of brite/beige adipocytes leads to an increase in mitochondrial uncoupling similar to that occurring in BAT. Two studies indicate that brite or beige adipocytes contribute significantly to whole body energy expenditure: mouse models that have increased beige/brite adipocytes in WAT are protected from diet-induced obesity (10), and browning of WAT contributes to non-shivering adaptive thermogenesis in the absence of classical BA (11). Adult humans possess both brown and beige/brite adipose tissue. Humans were thought to lose BAT after infancy, but phenotypically beige/brite adipocytes have been isolated from human supraclavicular fat depots and neck biopsies (9, 12, 13).

There is considerable interest in identifying additional agents that promote browning of adipose tissue, as increased expenditure of energy as heat would be of therapeutic utility in obesity and type 2 diabetes. To date, agents and processes with browning potential fall into a number of classes, namely (i) cold exposure, activation of the sympathetic nervous system (SNS), and β_3_-AR agonists; (ii) G protein-coupled receptor (GPCR), ion channel, and signaling pathway modulators; (iii) exercise and associated factors; (iv) growth factors and cytokines; (v) nutritional and dietary factors; and (vi) PPAR agonists. The evidence for these as browning agents has been reviewed elsewhere (14).

Many studies of browning agents have employed a combination of cultured adipocytes and whole animal experiments. Clearly *in vivo* experiments have the advantage that test compounds are acting on cell populations as they exist in whole animals, thereby providing valid information on predicted clinical efficacy. It is important to understand the mechanism of action, however, particularly in relation to the precise cells targeted by browning agents. In the whole animal, such agents could be acting directly on adipocytes, but it is equally possible that they are targeting the central nervous system (15), or indirectly the sympathetic nervous system (16).

Cultured adipocytes thus offer a system for characterizing the direct effect of browning agents, and also have advantages in facilitating high-throughput screening of compounds. The ideal model system would be cultured human adipocytes with the potential to undergo browning, however, there have been difficulties in using human cultures or cell lines—(i) even in human subjects with highly inducible BAT (17), beige/brite adipocytes are localized to the neck and supraclavicular regions, and presumably arise from specialized cells within these adipose depots, and (ii) human primary cultures or immortalized lines such as SGBS cells require strongly adipogenic media in order to differentiate, including, for example, rosiglitazone, dexamethasone, 3-isobutyl-1-methylxanthine (IBMX), cortisol, transferrin, triiodothyronine, and insulin (9, 18). In particular, the inclusion of rosiglitazone and IBMX (to increase cAMP), is highly likely to promote browning in conjunction with differentiation.

The primary cultured adipocytes most often utilized to study browning are differentiated from the stromal vascular fraction (SVF) of mouse inguinal WAT (iWAT) depots. Again these cultures generally include rosiglitazone at least for the first 2–4 days of culture (19–23), thus the mature adipocytes are likely to have undergone browning as well as differentiation. The aim of our study was to systematically clarify the effect of rosiglitazone on cultured adipocytes in the presence or absence of recognized browning agents targeting the β_3_-AR. We have examined adipocytes isolated from FVB/N mouse interscapular brown and inguinal white depots and cultured in a minimal medium consisting of DMEM supplemented with 10% newborn calf serum, 4.5 g/l glucose, and 2.4 nM insulin (8). We tested the effect of 1 µM rosiglitazone added to the culture medium for the entire 7 days or for the first 3 days only. The adipocyte cultures were treated for a further 24 h with CL316243 (in the absence of rosiglitazone), as a recognized browning agent. We find that BA cultures differentiate well even in the absence of rosiglitazone, whereas inguinal white adipocytes (iWA) require rosiglitazone for at least the first 3 days of culture. Substantial browning occurs only after 7-day rosiglitazone treatment in iWA, though induction of UCP1 and the thermogenic gene Cpt1b can be induced by CL316243 after 3 days of rosiglitazone. The highest levels of UCP1 mRNA occur following 7-day rosiglitazone combined with CL316243 treatment, and the vast majority of BA and iWA cells become positive for UCP1 immunostaining under these conditions.

## Materials and Methods

### Ethical Statement

All experiments were conducted with ethical permission from the Monash University Animal Ethics Committee, ethics approval numbers MIPS.2015.14 and VCP.2009.22, which complied with the National Health and Medical Research Council of Australia (NHMRC) guidelines for use of animals in scientific research.

### Adipocyte Culture

Adipocyte isolation and culturing was performed as described previously (24). Inbred FVB/N mice (3–4 weeks of age, either sex) were bred at the Monash University Parkville animal facility. Mice were killed by CO_2_ inhalation and BAT isolated from the interscapular, cervical, and axillary depots, while WAT was isolated from the subcutaneous depots. Pooled tissue pieces were finely minced in DMEM and transferred to a digestion solution [0.2% (wt/vol) collagenase type II, 0.1 M HEPES (pH 7.4), 123 mM NaCl, 5 mM KCl, 1 mM CaCl_2_, 4.5 mM glucose, 1.5% (wt/vol) BSA]. Tissues were digested for 30 min at 37°C with continuous mixing. Cells were filtered through a 250 µm nylon mesh filter into sterile tubes and kept on ice for 15 min whereupon the mature adipocytes float to the top. The top layer of the suspension was removed and the remaining cell suspension filtered through a 25-µm nylon mesh filter and centrifuged (700 × *g*, 10 min). The pellet containing preadipocytes was resuspended in DMEM and centrifuged (700 × *g*, 10 min). The pellet was suspended in culture medium (6 ml/animal for WAT, 4.8 ml/animal for BAT) and plated in either Seahorse cell culture plates (100 µl/well), 8-well chamber slides (200 µl/well), or 6-well plates (2 ml/well). The culture medium consisted of DMEM containing 4.5 g/l glucose, 10% (vol/vol) newborn calf serum, 2.4 nM insulin, 25 µg/ml sodium ascorbate, 10 mM HEPES, 4 mM l-glutamine, 50 U/ml penicillin, and 50 µg/ml streptomycin, and supplemented where indicated with 1 µM rosiglitazone. Adipocytes were grown at 37°C in 8% CO_2_. Cells were washed in pre-warmed DMEM and medium renewed on day 1, then every second day. All experiments were conducted on day 7. For experiments treated for 7 days with 1 µM rosiglitazone (7-day Rosi), rosiglitazone was included in control media from day 1 to 7 (the day of experiment). In some experiments as indicated, 1 µM rosiglitazone was only included in the culture media from day 1 to 3, whereupon the cells were cultured in the absence of rosiglitazone until use on day 7.

### Reverse Transcription-qPCR

Cells were serum starved on day 6 in DMEM/Nutrient Mix F-12 (1:1) with 4 mM l-glutamine, 0.5% BSA, 2.4 nM insulin, 10 mM Hepes, 50 IU/ml penicillin, 50 µg/ml streptomycin, and 50 µg/ml sodium ascorbate, with rosiglitazone (1 µM) as indicated. Media was replaced with DMEM containing 4.5 g/l glucose, 0.5% BSA, 25 µg/ml sodium ascorbate, 10 mM HEPES, 4 mM l-glutamine, 50 U/ml penicillin, and 50 µg/ml streptomycin, and supplemented with norepinephrine (1 µM), or CL316243 (1 µM) as indicated. Media was aspirated, the cells washed in warmed PBS, and plates rapidly frozen at −80°C until use. Total RNA was extracted using RNeasy Plus Mini Kits (QIAGEN), as per the manufacturer’s instructions (samples in Figure 5 were extracted using TriReagent (Sigma-Aldrich), according to the manufacturer’s instructions). RNA samples were DNAse treated using DNA*-free* DNA Removal Kit (Invitrogen), according to manufacturer’s instructions. Where tissues were used, interscapular BAT or inguinal WAT were excised from similarly aged (3- to 4-week-old FVB/N mice of either sex) and housed mice (room temperature 22°C), and tissues rapidly frozen (−80°C). RNA from tissues was extracted using TriReagent according to manufacturer’s instructions (Sigma-Aldrich).

For preparation of cDNA, 0.5 µg of RNA was reverse-transcribed using iScript Reverse Transcription Supermix for RT-qPCR (Bio-Rad), in a total volume of 10 µl. For each independent sample, qPCR was performed in duplicate using TaqMan Gene Expression assays (Life Technologies) for Prdm16, Hoxc9, Ppara, Ppargc1a, Pparg, Ppargc1b, Fabp4, Adipoq, Slc27a1, Fasn, Pck1, Acaca, Fabp3, Acox1, Sirt3, Cpt1b, Cox4i1, Prdx3 Acadl, Vdac1, Pdk4, Slc2a1, Slc2a4, Hk2, Pfkm, Gapdh, Pgk1, Ucp1, Adrb3, and the reference gene Actb. The cDNA was diluted to the equivalent of 2.5 ng/µl of starting RNA and 4 µl added to 6 µl reaction mix comprising 1× TaqMan Gene Expression Assay and 1× TaqMan Fast Advanced Master Mix dispensed in 96-well plate, as per manufacturer’s instructions. After initial denaturation at 95°C for 30 s, fluorescence was detected over 40 cycles (95°C for 5 s, 60°C for 30 s). C_q_ values were automatically calculated by the Realplex analysis module. qPCR presented in Figure 5 was performed on a CFX Connect™ real-time PCR detection system (BioRad) and samples were initially denatured at 50°C for 2 min, 95°C for 10 min, and fluorescence detected over 40 cycles (95°C for 15 s, 60°C for 1 min), and C_q_ values automatically calculated by the BioRad analysis module. All data are expressed as expression of the gene of interest relative to Actb, calculated as $(2^{-{\Delta \text{C}}_{\text{q}}})*1,000$. Multiplication of all values by 1,000 does not change the relative expression levels and was done for two reasons; (i) it facilitates viewing of the data because even values for poorly expressed genes are greater than 1.0, and (ii) it positions expression values in the same range as RPKM or FPKM values obtained in RNA-Seq studies, as the average value for Actb in numerous cell and tissue types is 1,000. All statistics for gene expression were performed on ΔC_q_ values, as these data are normally distributed. For the purpose of statistics, where genes were not detected within 40 cycles (and were, therefore, not detectably expressed), an over-assumption C_q_ value of 40 was used.

### Custom PCR Array

We designed a custom mouse qPCR array in 384-well plates that comprised 2 sets of 192 genes, including Actb as a reference gene, a genomic DNA control (Lonza) and 190 genes representing a broad cross-section of targets downstream of 25 different transcription factors (Table 1). Each array plate was used to analyze expression in a control and rosiglitazone-treated adipocyte culture from BA or iWA. cDNA was prepared as described above, then 4 µl equivalent to 10 ng of starting RNA was added to each well containing 1× SYBR Green PCR Master Mix (Applied Biosystems) and Lonza-dispensed primer sets in a total volume of 10 µl. qPCR reactions were carried out at the Australian Genome Research Facility (Parkville, VIC, USA) on a 7900HT Real-Time PCR System (Applied Biosystems).

**Table 1:** Categorization of genes assessed by the StellARray custom array system.

Gene-associated category	Genes
Signaling	
Kinases	*Akt1, Camkk1, Map2k2, Map3k7, Pak2, Pyk2, Trib3*
Phosphatases	*Dusp1, Dusp9, Ppm1d, Ppp1r15a, Ppp3ca, Pten*
G Protein-related	*Arhgef2, Rgs2*
Regulatory binding proteins	*Pmepa1, Rcan1*
Apoptosis, stress response	*Bax, Bbc3, Bcl10, Bcl2, Bcl2l1, Bcl2l11, Birc3, Birc5, Bnip3, Bnip3l, Casp1, Casp3, Casp9, Cflar, Fas, Gadd45a, Gadd45b, Hspa1a, Hspa1b, Hspa5, Hspb1, Hspb2, Prdx3, Sp1, Xiap*
Cell cycle regulation	*Ccna2, Ccnd1, Ccnd2, Ccnd3, Ccne1, Ccne2, Cdk2, Cdk4, Cdkn1a, Cdkn1b, Cdkn1c, Cdkn2a, Cdkn2b, Lats2, Rb1, Sik1, Trp53*
Cytoskeletal constituents and reorganization	*Acta1, Acta2, Actb, Cap1, Grasp, Hsp90b1, Rac1, Vcl*
Proteolysis, ubiquitination	*Fbxo32, Mdm2, Socs1, Socs3*
Transcription factors, transcriptional regulators	*Atf3, Bcl6, Calr, Cebpb, Ddit3, Ddit4, Egr1, Esrra, Fos, Hdac1, Hdac9, Hes1, Jun, Junb, Klf10, Mef2a, Mef2c, Myc, Nfkb1, Nfkbia, Notch1, Nr4a3, Per2, Pparg, Ppargc1a, Ppargc1b, Srebf1, Srf, Stat1, Stat3, Stat6*
Enzymes	*Acaca, Acadl, Acox1, Cox4i1, Cpt1a, Cpt1b, Gamt, Gapdh, Gck, Gls2, Gsk3b, Hk2, Hmox1, Ldha, Lpl, Mest, Nos2, Pck2, Pcx, Pfkm, Ptgs2, Sod1, Sod2, Sod3*
Hormones, growth factors, cytokines	*Adipoq, Angpt1, Angpt2, Angptl4, Bmp4, Ccl2, Ctgf, Igf2, Il6, Inhba, Nppb, Ptn, Rspo1, Smad7, Spp1, Tgfb1, Tnf, Vegfa*
Mitochondrial function	*Fasn, Lonp1, Sirt3, Tfam, Tomm20, Ucp2, Ucp3, Vdac1*
Ion channels, transmembrane transporters	*Fabp3, Slc2a1, Slc2a4*
Extracellular matrix, cell adhesion	*Col1a1, Icam1, Mmp9, Mmp13, Serpine1, Vcam1*
Additional genes in array with negligible adipocyte expression	*Alox5, Bmp2, Cap2, Ccl26, Ccna1, Ccnb1, Cdk1, Dapk2, Foxa2, Gata4, Hey2, Ins1, Ins2, Isl1, Kcnj11, Mafa, Mmp7, Mstn, Myocd, Myog, Neurod1, Nkx2-2, Nkx2-5, Nkx6-1, Nr4a1, Nr4a2, Pdx1, Ppp1r3a, Slc2a2, Xirp2*

### Ingenuity Pathway Analysis (IPA)

Patterns of gene expression were examined using IPA software. For each of the adipocyte datasets, we uploaded fold-change between rosiglitazone treated and control cultures, along with the final expression value in rosiglitazone-treated cultures and *P* values for significant differential expression. The datasets were filtered for fold-changes of at least 2 in either direction, expression values normalized to Actb (as described above) of at least 1.0 in at least one culture condition, and *P*-values <0.05 (Student’s *t*-test), leaving 171 genes. IPA core analysis was used to determine enrichment of molecular and cellular functions or upstream regulators predicted from observed patterns of up or down-regulation among the expressed genes. IPA uses Fisher’s Exact Test to test the hypothesis that patterns of gene expression related to functions or to upstream regulators are not due to chance. This analysis provides *P* values that signify the degree of enrichment of differentially expressed genes, and activation *z*-scores that take into account the direction of change compared to effects predicted from the IPA knowledge base (25). A *z*-score of at least 2.0 is considered significant.

### External Data

We reanalyzed publically available RNA sequencing data obtained from Hao et al. (26) (upstream analysis shown in Figure 6). In this published study 8-week-old male C57BL/6J mice were housed at thermoneutrality (28–30°C) on a 12:12 h light–dark cycle and fed standard chow diet *ad libitum*. After 8 days, mice were either retained at thermoneutrality or transferred to 4°C for 2 or 4 days. Mice were killed by cervical dislocation for isolation of total RNA from interscapular BAT and inguinal WAT. Data consisting of tag counts per million were generated by digital gene expression profiling, mapped to NCBI RefSeq mRNAs, and deposited in Gene Expression Omnibus (accession number GSE63031). We uploaded the 0- and 2-day cold exposure datasets to IPA to compare upstream regulators between BAT and iWAT, as described in the legend to Figure 6. The mouse experiments described by Hao et al. (26) were approved by the Norwegian or Danish Animal Research Authority.

### Immunocytochemistry for Detection of UCP-1

Cells were seeded in 8-well culture chamber slides (BD Biosciences, Franklin Lakes, BJ). On day 7, cells were fixed with 4% formaldehyde in PBS for 15 min, and quenched with 150 mM Tris pH 8.0 for 10 min. Cells were permeablized with 0.1% Triton X-100 for 10 min, blocked for 1 h at room temperature (5% BSA in PBS), and incubated with UCP1 (Abcam) primary antibody solution (diluted 1:1,000 in 1% BSA in PBS) overnight at 4°C. Alexa Fluor 488-conjugated goat anti-rabbit IgG (1:1,000 dilution in 1% BSA in PBS) was added and incubated at room temperature for 2 h. Images were observed on a Leica DMLB epifluorescence microscope. Images were acquired using a DC350F camera with IM500 software (Leica Microsystems AB; Kista, Sweden). Quantification was performed using Fiji (ImageJ version 1.50 g) (27), using a script developed by C.J. Nowell. Cells identified by DAPI staining in images were judged to be negative/positive for the protein of interest (UCP-1) in a blinded manner and counted using ImageJ software, performed by J. Merlin. No distinction was made in the relative intensities of staining within images.

### Measurement of Oxygen Consumption Rates

Oxygen consumption rates (OCR) were measured using the Seahorse xF96 (Seahorse Bioscience). On day 7, adipocytes were washed twice in XF assay media (Seahorse Bioscience) supplemented with 25 mM glucose, 0.5 mM sodium pyruvate, 2 mM l-glutamine and 1% fatty free BSA, and 160 µl added/well. OCR was measured as described in detail (28) with some modifications. 6 baseline rate measurements were made using a 2 min mix, 5 min measure cycle. After 6 basal measurements, oligomycin A (5 µM) was added for 6 measurements, followed by a combination of antimycin A (1 µM) and rotenone (0.1 µM) for 6 measurements. OCR rates immediately prior to oligomycin A injection at measurement number 6 were used as the basal rates and defined as 100%.

### Statistical Analysis

All data are expressed as mean ± SEM of *n*. Statistical significance was determined by Student’s *t*-test, multiple comparisons one-way ANOVA with Tukey’s test, or multiple comparisons Kruskal–Wallis test (for non-parametric analyses), as indicated in Figure legends. *P* < 0.05 was considered significant.

## Results

### Rosiglitazone Treatment of Cultures From Inguinal WAT Increases Differentiation and the Expression of Genes Related to Thermogenesis

**Figure 1:**
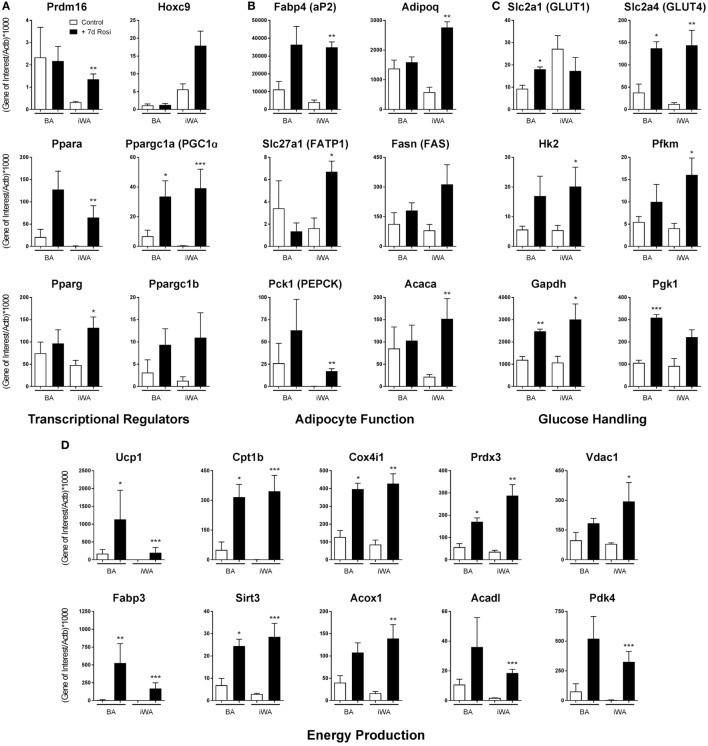
Rosiglitazone treatment increases expression of genes associated with thermogenesis in white adipocyte cultures. 7-day 1 µM rosiglitazone treatment (7-day Rosi) of brown (BA), or inguinal white adipocyte (iWA) cultures derived from the stromal vascular fraction, increased the expression of a range of transcriptional regulators **(A)**, adipocyte function genes **(B)**, genes involved in the uptake and handling of glucose **(C)**, and genes involved in adipocyte energy production **(D)**. Data represent mean ± SEM of three independent experiments, performed in duplicate, relative to β-actin expression. *P < 0.05, **P < 0.01, ***P < 0.001 indicate statistical significance (unpaired Student’s *t*-test) between rosiglitazone-treated and control cultures.

7-day rosiglitazone treatment (1 µM) promoted expression of genes associated with both maturation of white adipocytes, and thermogenic re-programming (Figure 1; Table 2). While BA cultures differentiate in the absence of rosiglitazone, displaying robust expression of the mature adipocyte marker Fabp4 (Figure 1B), iWA cultures express lower levels of Fabp4 (36% relative to BA), indicating that a much lower proportion of cells undergo differentiation under control conditions (in DMEM containing 4.5 g/l glucose, 10% (vol/vol) newborn calf serum, and 2.4 nM insulin). However, following 7d of rosiglitazone treatment, Fabp4 mRNA levels are similar to that in iWAT tissues, and also to BA tissue and cultures (Figure 1; Table 2). Likewise, the genes Acaca (acetyl CoA carboxylase), Fasn (fatty acid synthase), Slc27a1 (FATP1), Pck1 (phosphoenolpyruvate carboxykinase), and Adipoq (adiponectin), all characteristic of mature adipocytes, are expressed in control and treated BAs, whereas all of these genes are low in control iWA, and significantly increased by rosiglitazone (Figure 1B). Conversely, the white adipocyte marker Hoxc9 is expressed to some extent in both control and rosiglitazone-treated iWA cultures but is negligible in BA under any conditions (Figure 1).

**Table 2:** Comparison of gene expression in interscapular brown (BA) and inguinal white adipocytes (iWA) cultured for 7 days in the presence or absence of rosiglitazone (Rosi, 1 µM, 7 days), and in freshly isolated brown adipose tissue (BAT) and iWAT from mice housed at 22°C.

Gene	Cultured BA (control)	Cultured BA (+Rosiglitazone)	Interscapular BAT
Acot11	1.58 ± 0.34	2.58 ± 1.24	396 ± 65.6
Cpt1b	49.7 ± 39.9	317 ± 64.5	607 ± 97.3
Fabp3	6.94 ± 6.12	527 ± 272	123 ± 32.7
Fabp4	11,223 ± 4,563	36,435 ± 10,226	10,179 ± 2,555
Sirt3	17.1 ± 9.85	59.1 ± 13.4	79.3 ± 17.2
Ucp1^a^	2.62 ± 0.57	982 ± 375	10,344 ± 3,271
Prdm16	2.34 ± 1.35	2.17 ± 0.66	11.3 ± 1.49
Ppargc1a	6.87 ± 4.09	33.6 ± 10.6	22.3 ± 1.25
Pck1	26.2 ± 22.3	63.1 ± 34.8	1,115 ± 168
Pdk4	96.3 ± 84.9	521 ± 284	819 ± 157
Sirt1	8.42 ± 3.83	8.29 ± 1.15	9.06 ± 1.02
Adrb3^a^	3.38 ± 1.49	4.23 ± 0.99	4.19 ± 1.24

**Gene**	**Cultured iWA (control)**	**Cultured iWA (+Rosiglitazone)**	**Inguinal WAT**

Acot11	0.21 ± 0.06	0.91 ± 0.08	12.8 ± 0.78
Cpt1b	0.76 ± 0.21	346 ± 80.9	104 ± 13.9
Fabp3	0.19 ± 0.03	169 ± 79.7	63 ± 14
Fabp4	4070 ± 1211	34890 ± 3021	12,144 ± 2,412
Sirt3	2.99 ± 0.71	47.3 ± 12	18.1 ± 2.36
Ucp1^a^	0.04 ± 0.03	45.9 ± 13.4	1,775 ± 361
Prdm16	0.33 ± 0.04	1.35 ± 0.24	2.63 ± 0.41
Ppargc1a	0.47 ± 0.06	39.2 ± 12.8	7.74 ± 2.3
Pck1	0.26 ± 0.12	17.3 ± 2.71	419 ± 43.2
Pdk4	5.52 ± 0.59	326 ± 88	139 ± 23.8
Sirt1	4.69 ± 0.15	8.04 ± 1.16	4.65 ± 0.59
Adrb3^a^	0.49 ± 0.26	3.32 ± 1.01	3.04 ± 0.17

^a^Data for Ucp1 and Adrb3 expression in control and rosiglitazone-treated adipocytes obtained from Merlin et al. (29). All data are expressed relative to Actb expression*1,000 ± SEM of three independent experiments (as per Figure 1).

The BA transcriptional regulator Prdm16 is expressed in both control and rosiglitazone-treated BA cultures, and is substantially increased in iWA cultures treated with rosiglitazone (Figure 1A), suggesting browning of iWA under 7-day rosiglitazone treatment as we have shown previously (29). Pparg, encoding the rosiglitazone-targeted nuclear receptor PPARγ, a major regulator of adipocyte differentiation, is expressed in control white adipocyte cultures but also significantly increased by rosiglitazone in treated iWA. These findings indicate that control iWA cultures contain few fully differentiated adipocytes, but a significant population of Hoxc9-positive preadipocytes (Figure 1A). Upon rosiglitazone treatment, levels of Fabp4, Adipoq, Fasn, Slc27a1, and Acaca expression in iWA equal or exceed those found in treated BA cultures, indicating a high degree of differentiation.

Figure 1D shows genes involved in fatty acid metabolism and mitochondrial function. In particular, Cpt1b (carnitine palmitoyltransferase 1B) is the rate-limiting step for fatty acid oxidation and consequent stimulation of mitochondrial respiration, while Fabp3 is also required for efficient fatty acid oxidation (30). Cpt1b mRNA is 65 times higher in control BA than in iWA cultures, and in BA, undergoes 6.4-fold induction in the presence of rosiglitazone. iWA display a striking 455-fold induction, reaching levels similar to those in BA. Similarly, Fabp3 is induced by 630-fold in iWA cultures following rosiglitazone treatment. Additional genes related to thermogenesis (Acadl, Vdac1, Acox1, Sirt3, Pdk4, Prdx3, Cox4i1) display significantly higher expression in rosiglitazone-treated iWA than in control cultures. Ppara (PPARα), which promotes expression of genes essential for many aspects of fatty acid metabolism, and Ppargc1a (PGC-1α), a master regulator of adipocyte browning and mitochondrial biogenesis, are induced by 77- and 83-fold, respectively, in adipocytes from iWAT following rosiglitazone treatment (Figure 1A).

Analysis of genes related to glucose handling (Figure 1C) showed significant increases in the insulin responsive GLUT transporter Slc2a4 (GLUT4), and glucose metabolism enzymes Hk2, Pfkm, and Gapdh, in iWA cultures treated with rosiglitazone, further indicating the differentiation of iWA cultures toward an insulin-sensitive mature adipocyte population. Rosiglitazone treatment also increased the expression of Slc2a4, Gapdh, and Pgk1 in BAs, likewise suggesting changes in insulin-mediated glucose handling, but also a significant increase in Slc2a1 (GLUT1), which has been shown to be the major GLUT involved in β_3_-AR-mediated glucose uptake in BA (31).

**Figure 2:**
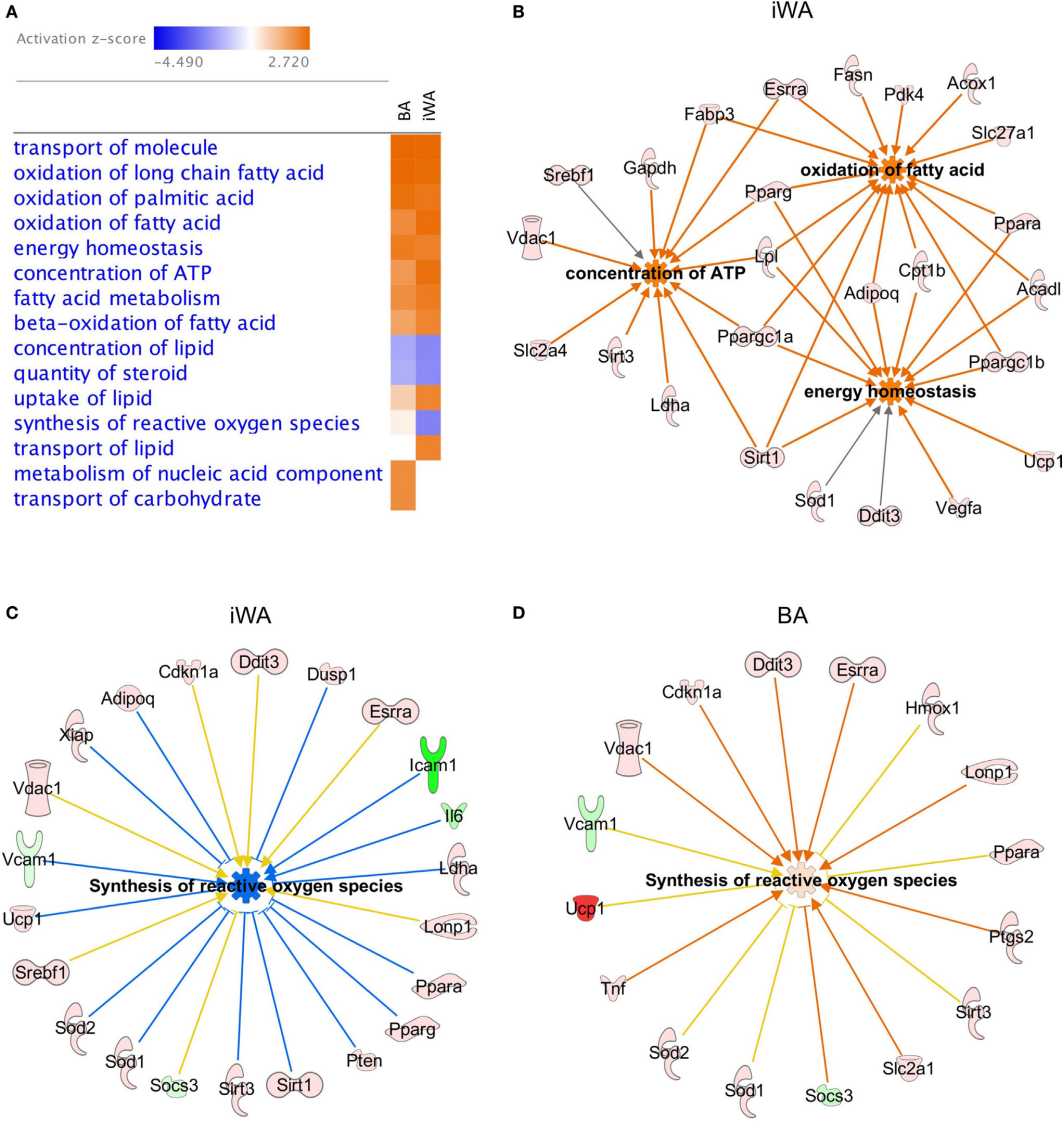
Molecular and cellular functions of genes differentially expressed in control or rosiglitazone-treated adipocytes. Ingenuity Pathway Analysis (IPA) software was used to compare downstream functions consistent with observed patterns of target gene up- or down-regulation in adipocyte cultures treated with rosiglitazone relative to vehicle **(A)**. Functions are ranked according to the sum of activation *z*-scores across brown adipocyte (BA) and inguinal white adipocytes (iWA) cultures, irrespective of positive or negative direction. *Z*-scores ≥|2| describe statistically significant matches between known functions and observed patterns of gene regulation, and also whether functions are predicted to be up- or down-regulated downstream of the observed changes (activated shown in orange, inhibited shown in blue) (25). Network of genes upregulated in iWAT that contribute to key non-redundant cellular functions (oxidation of fatty acid, concentration of ATP and energy homeostasis) **(B)**. Differentially expressed genes identified by IPA that contribute to synthesis of reactive oxygen species (ROS) in iWA **(C)** and BA **(D)**. In both panels, coloring of genes red or pink denotes upregulation and green denotes downregulation. In panel **(C)**, blue lines signify genes showing a change in expression consistent with reduced ROS synthesis, while yellow lines signify genes that are not consistent with this function. The *z*-score for iWA is −2.2, below the significance threshold of |2|, whereas that for BA is +0.27 and not significant. iWA cultures treated with rosiglitazone display fourfold increases in expression of the key anti-oxidant genes Sod1 and Sod2 relative to control cultures.

In parallel with individual qPCR assays, we obtained a comprehensive view of rosiglitazone-induced changes in cultured mouse adipocytes by analyzing mRNA levels of 160 expressed genes quantified using a custom qPCR array (categorized in Table 1). Products of these genes participate in a broad cross-section of molecular and cellular functions, and represent targets of diverse upstream transcriptional regulators. We assessed the expression of 4 genes from both the qPCR array and TaqMan assays (Cpt1b, Fabp3, Ppargc1a, and Sirt3). Because expression is normalized to the reference gene Actb, the two methods give closely corresponding values for a given sample, showing less inter-assay variation than the inter-sample differences seen using either method. Genes displaying greater than twofold change of expression in either direction (*P* < 0.05) were subject to enrichment analysis using IPA software (Qiagen). The significantly enriched molecular and cellular functions are similar between BA and iWA (Figure 2A). The top score is for molecular transport (which encompasses a wide array of cellular processes), but the remaining significant functions are related largely to energy homeostasis, fatty acid metabolism, and ATP generation. Figure 2B shows the upregulated genes expected to contribute to these functions in rosiglitazone-treated iWAs.

As shown in Figure 2A, there are four functions that show an apparent difference between BA and iWA. Increases in the “transport of lipid,” “metabolism of nucleic acid component,” and “transport of carbohydrate” functions are significant in only one of iWA or BA, but in each of these three cases the number of genes showing a direction of expression change consistent with increased function is small. For example, significantly increased transport of carbohydrate is predicted in BA, based on enhanced expression of 10 genes positively linked to this function (Hk2, Ppargc1a, Ppargc1b, Ptgs2, Slc2a1, Slc2a4, Sod1, Tnf, Trib3, and UCP1). In iWA, only seven of these genes display increased expression, so in these cells transport of carbohydrate is no longer predicted to be significantly affected. This finding is not borne out by the functional phenotype of iWA, as rosiglitazone treatment significantly increases glucose uptake in response to norepinephrine and CL316243 (29). This reflects our observation that the limiting step in agonist-stimulated glucose uptake is the abundance of the β_3_-AR, which is markedly enhanced when iWAs are cultured in the presence of rosiglitazone [Table 2; (29)]. Interestingly, the function “synthesis of reactive oxygen species” is predicted to be decreased in iWA (*z*-score of −2.2, based on 23 differentially expressed genes), while this function is not significantly affected by rosiglitazone treatment in BA (*z*-score of +0.27, 16 genes). Genes displaying changes in expression expected to enhance or inhibit synthesis of reactive oxygen species (ROS) are shown in Figure 2C (iWA) and Figure 2D (BA). The overlapping genes all display the same direction of change in expression in iWA and BA, but in iWA cultures there are 15 genes for which up- or down-regulation is expected to reduce ROS. The induction of genes encoding anti-oxidant enzymes, including superoxide dismutase SOD1 and SOD2 in iWAT also occurs *in vivo* following rosiglitazone treatment (32).

### Brite Adipocytes Display Increased UCP1 Protein and Uncoupling Capacity in Response to 24 h Treatment With the β_3_-AR Agonist CL316243

**Figure 3:**
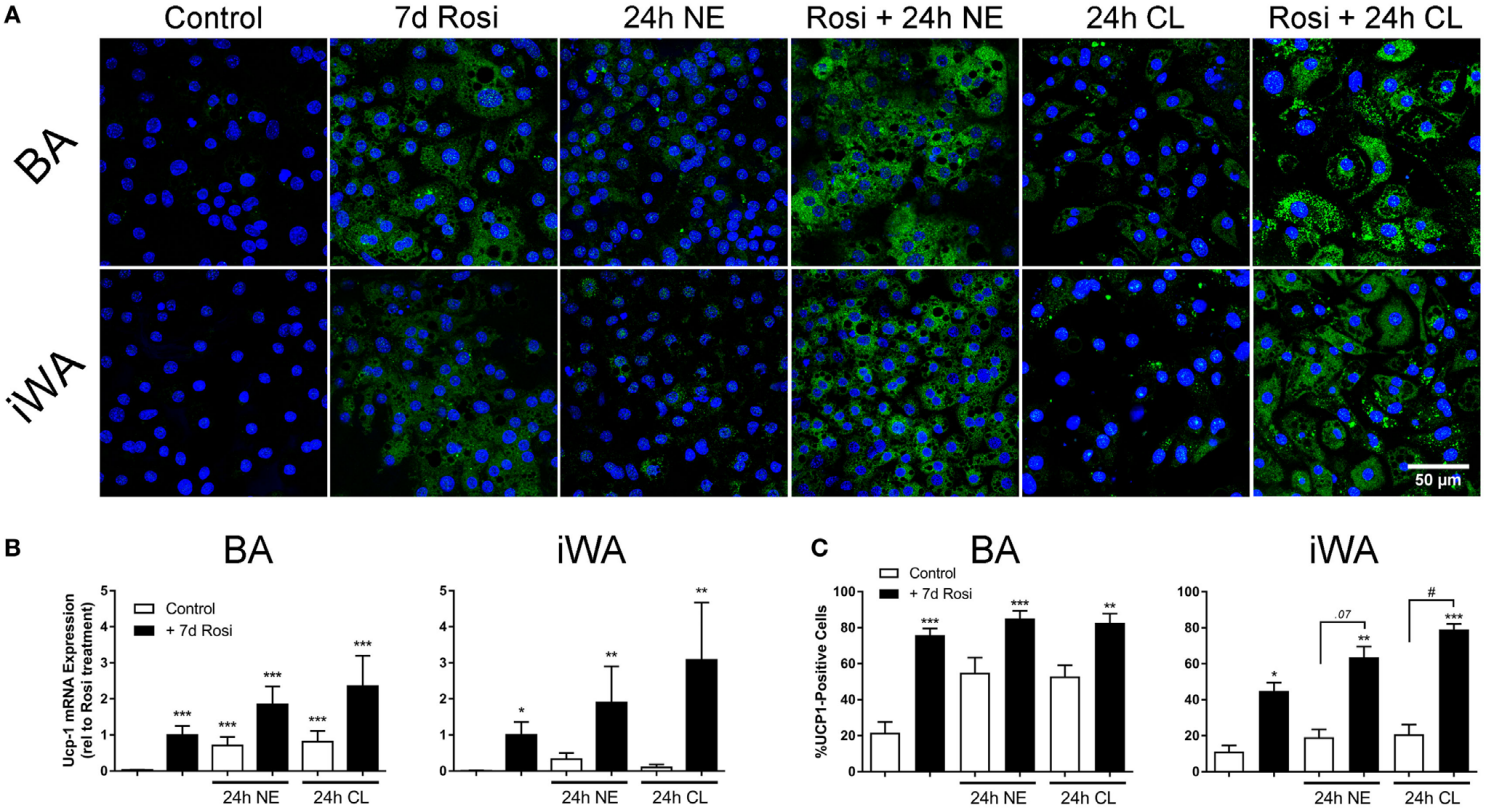
Effect of combined rosiglitazone and β-adrenoceptor agonist treatment on uncoupling protein 1 (UCP1) mRNA and protein content. **(A)** Representative images obtained using a UCP-1 antibody (green) in brown (BA) and inguinal white adipocytes (iWA) following rosiglitazone (1 µM, 7 days) treatment in the presence/absence of norepinephrine (NE, 1 µM, 24 h) or CL316243 (CL, 1 µM, 24 h). Nuclei are stained blue by DAPI staining. **(B)** Expression of UCP1 mRNA in BA and iWA following rosiglitazone (1 µM, 7 days) treatment in the presence/absence of norepinephrine (NE, 1 µM, 24 h) or CL316243 (CL, 1 µM, 24 h). Values from each rosiglitazone-treated BA experiment were expressed relative to their normalized value, and levels in all other cells/treatments expressed relative to this value, expressed as mean ± SEM from 3 to 5 independent experiments performed in duplicate. *P < 0.05, **P < 0.01, ***P < 0.001 indicate statistical significance from control (unpaired one-way ANOVA, Tukey’s multiple comparisons post-test). **(C)** Quantification of proportion of UCP-1-positive cells in **(A)**. Data represent mean ± SEM of 5–6 independent experiments. Data are confined between 0 and 100% and are, therefore, non-parametrically distributed. *P < 0.05, **P < 0.01, ***P < 0.001 indicate statistical significance from control cells, ^#^*P* < 0.05, ^##^*P* < 0.01 indicate statistical significance from respective adrenergic control (multiple comparisons Kruskal–Wallis test, one-way ANOVA).

As chronic β-AR treatment of BA upregulates UCP-1 mRNA and protein levels (1), we treated BA and iWA cultures in the presence/absence of 7-d rosiglitazone (1 µM) with norepinephrine (1 µM, 24 h) or CL316243 (1 µM, 24 h). Norepinephrine and CL316243 caused a small increase in UCP1 mRNA expression (Figure 3B) and immunoreactivity (Figures 3A,C) in control BA, but this response was significantly amplified in rosiglitazone-treated BA cultures. The effect of combined rosiglitazone and β-AR agonist treatment was even more evident in iWA cultures. These data are consistent with the presence or absence of β_3_-ARs in these cultures (29). There were significant changes in the proportion of UCP1-positive cells between all control cultures and those treated with rosiglitazone alone (Figures 3A,C). Following an additional 24 h in the presence of CL316243, there were also significant differences in UCP1-positive cells between control and rosiglitazone-treated iWA cultures (Figure 3C).

**Figure 4:**
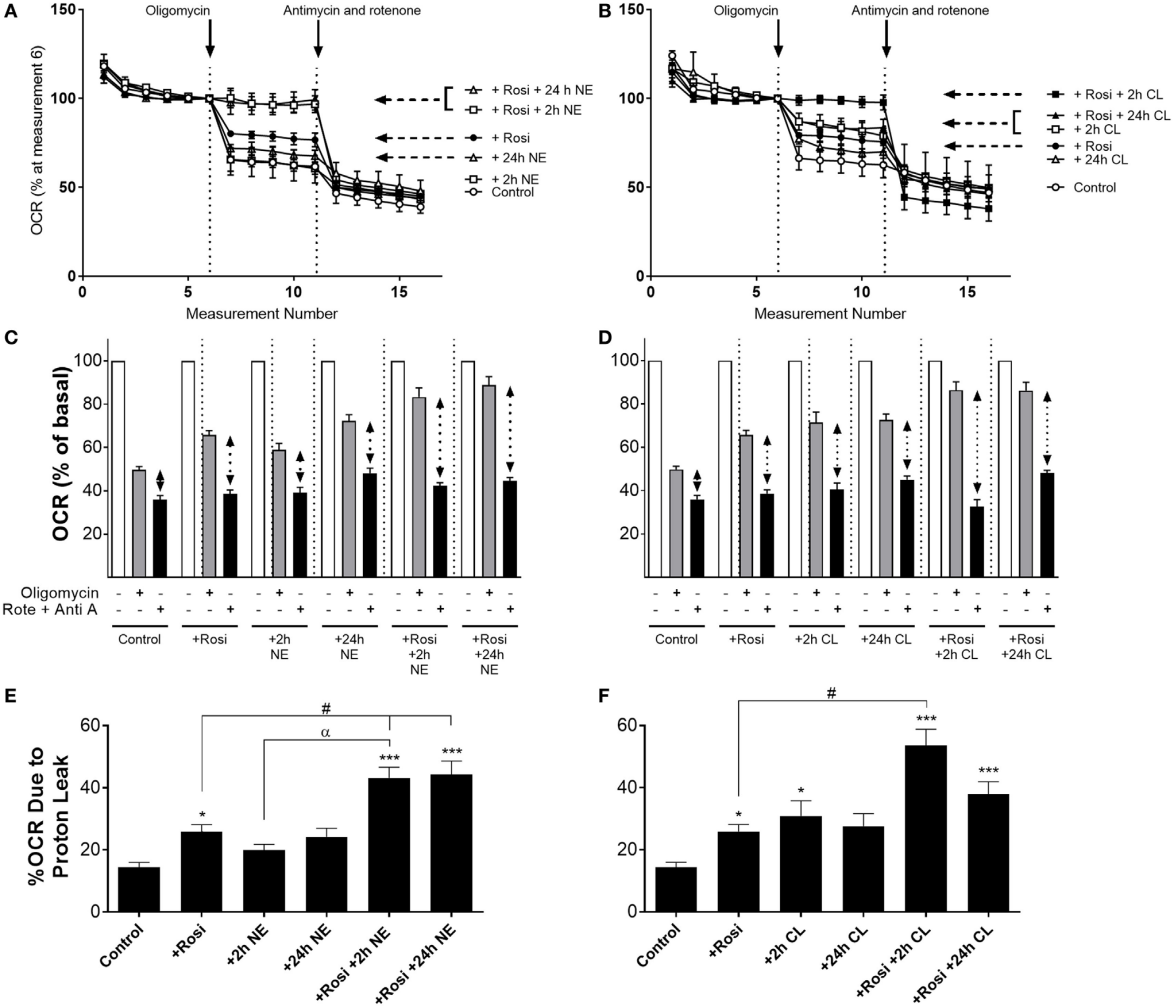
Effect of combined rosiglitazone and β-adrenoceptor agonist treatment on oligomycin-insensitive oxygen consumption in inguinal white adipocytes (iWA). Control or rosiglitazone (1 µM, 7 days) treated iWA were treated with **(A,C)** 1 µM norepinephrine (NE; 2 or 24 h) or **(B,D)** 1 µM CL316243 (CL; 2 or 24 h) prior to measurement of oxygen consumption rates (OCR). Cells were treated in the Seahorse XF96 with the ATP-synthase inhibitor oligomycin (5 µM) or a combination of the mitochondrial inhibitors 0.1 µM rotenone (Rote) and 1 µM antimycin A (Anti A). Data are mean ± SEM of 12–38 independent experiments performed in duplicate. Data in **(A,B)** are representative traces of two experiments performed in duplicate. Arrows indicate the addition of oligomycin (5 µM) or the combination of rotenone (0.1 µM) and antimycin A (1 µM), with basal OCR set to 100% before the addition of oligomycin at rate 6 to account for variations in the raw data between adipocyte cultures made on different days. **(E,F)** The relative changes in OCR between oligomycin (defining OCR due to ATP synthase) and rotenone/antimycin A (defining OCR due to non-mitochondrial sources) are expressed as % OCR due to proton leak [calculated from the results presented in **(C,D)** indicated with the dotted arrows]. Data are non-parametrically distributed and, therefore, statistically analyzed by non-parametrically analysis. *P < 0.05, **P < 0.01, ***P < 0.001 indicate statistical significance from control cells, ^#^*P* < 0.05 indicates statistical significance from rosiglitazone-treated cells, ^α^*P* < 0.05 indicates statistical significance from adrenergic treatment alone (multiple comparisons Kruskal–Wallis test, one-way ANOVA).

In order to investigate whether the increase in UCP1 mRNA/protein levels following rosiglitazone, norepinephrine, or CL316243 treatment affected mitochondrial uncoupling in white adipocytes, we measured OCR in the presence of the ATP synthase inhibitor oligomycin (5 µM), and in the presence of rotenone (0.1 µM) and antimycin A (1 µM), that define non-mitochondrial sources of OCR. In control iWA cells, oligomycin inhibited OCR by ~50% (Figure 4). In iWA cells treated 7-day with rosiglitazone (1 µM), oligomycin inhibited OCR by only 34%. The insensitivity to oligomycin was enhanced when rosiglitazone treated cells were also treated with either NE (1 µM, 2 and 24 h) or CL316243 (1 µM, 2 and 24 h). This suggests an increase in uncoupled respiration. This was further assessed by treating the cells with a combination of rotenone (0.1 µM) and antimycin A (1 µM), that define non-mitochondrial sources of OCR (~36% of OCR was due to non-mitochondrial sources; Figure 4). After correction for non-mitochondrial OCR (indicated in dotted arrows in Figures 4C,D, and illustrated as % OCR due to proton leak in Figures 4E,F), we can see that mitochondrial uncoupling (proton leak) accounts for 14% of OCR in control iWA cells, 27% in rosiglitazone-treated cells, and this level rises to 40–50% in rosiglitazone-treated iWA cells further treated with NE or CL316243. These results suggest that rosiglitazone-induced brite adipocytes possess a greater mitochondrial uncoupling capacity, and that β_3_-AR treatment of brite adipocytes significantly increases this capacity.

### The Effects of Rosiglitazone on Adipocyte Differentiation, and the Thermogenic Potential of iWA, Are Separable Following 3-Day Treatment

**Figure 5:**
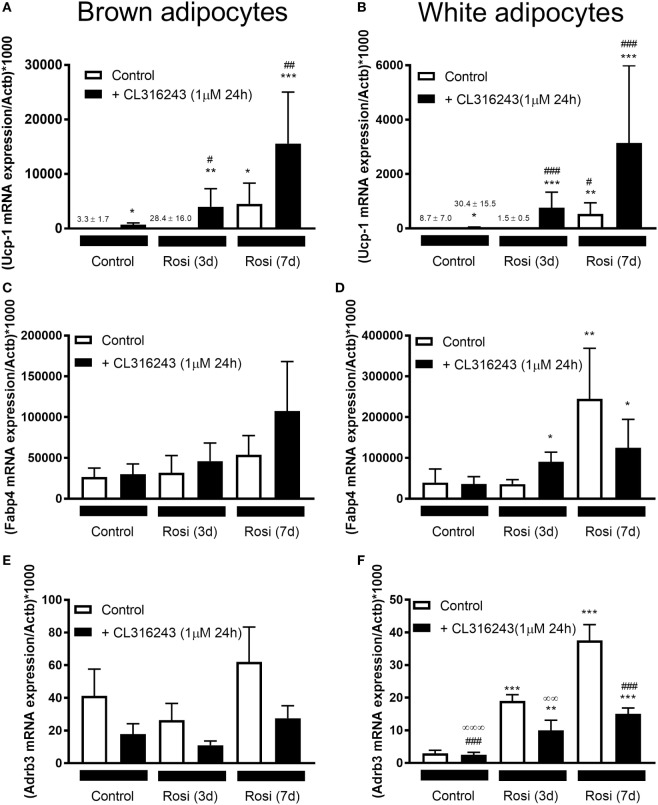

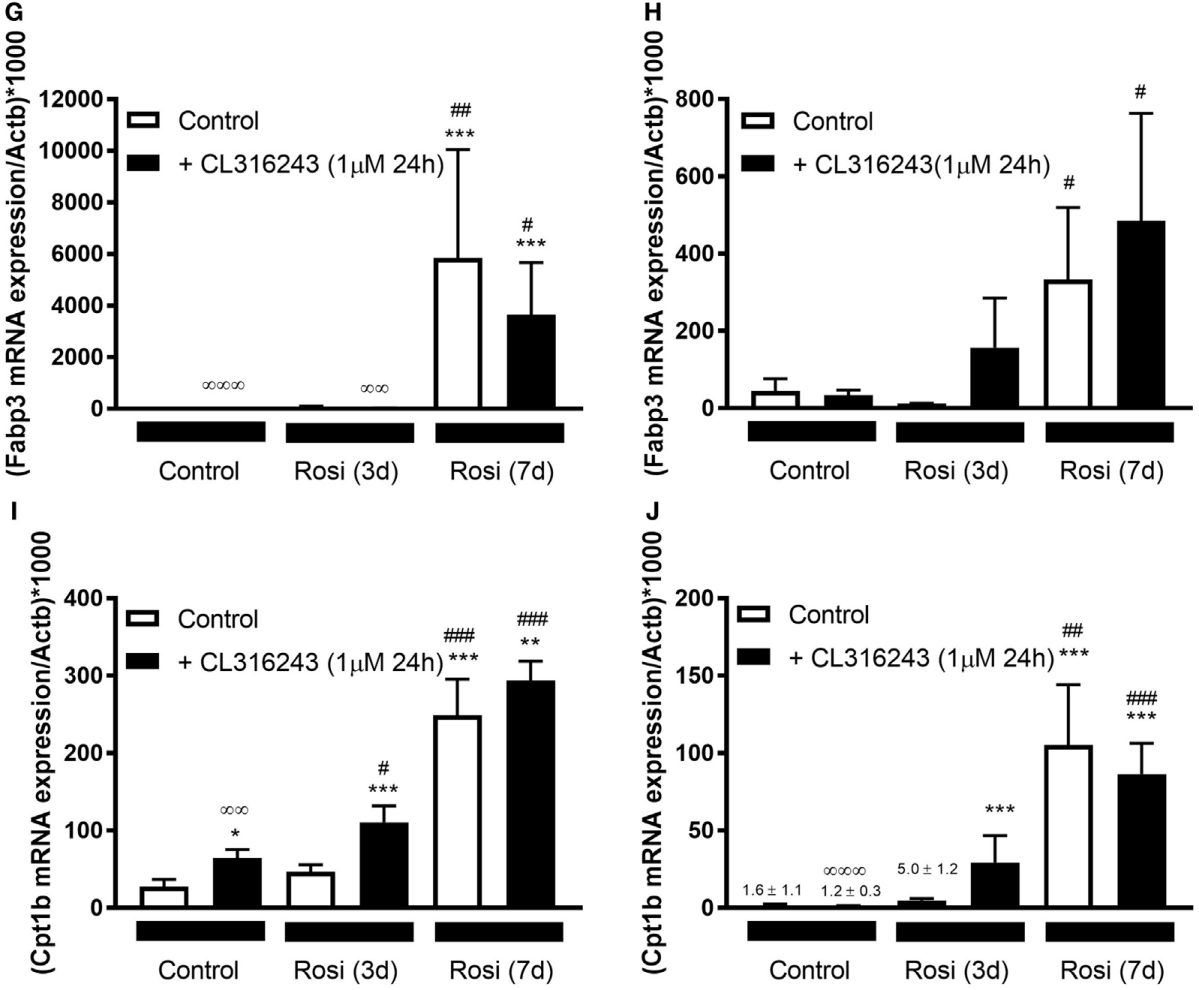
Effect of different lengths of time of rosiglitazone on gene expression in white and brown adipocytes (BA). Control or rosiglitazone [1 µM, 3 days (3d), or 7 days (7d)] treated brown or inguinal white adipocytes (iWA) were treated with 1 µM CL316243 (24 h) prior to measurement of several genes, including Ucp1 **(A,B)**, Fabp4 **(C,D)**, Adrb3 **(E,F)**, Fabp3 **(G,H)**, or Cpt1b **(I,J)**. Data represent mean ± SEM of 6 (BA) or 7–8 (iWA) independent experiments, performed in duplicate, relative to β-actin (Actb) expression. *P < 0.05, **P < 0.01, ***P < 0.001 indicate statistical significance between all treatments and the control cultures. ^#^*P* < 0.05, ^##^*P* < 0.01, ^###^*P* < 0.001 indicate statistical significance between all treatments and the Rosi (3d) treated cultures. ^∞^*P* < 0.05, ^∞∞^*P* < 0.01, ^∞∞∞^*P* < 0.001 between all treatments and the Rosi (7 days) treated cultures. Data analyzed by Tukey’s multiple comparisons of one-way ANOVA performed on the ΔC_t_ values, which are normally distributed. One sample (iWA treated with rosiglitazone for 3 days) was excluded from all analysis due to poor integrity of its RNA. A single measurement of *Adrb3* for iWA treated with CL316243, and of Fabp4 for BA treated with Rosi 7 days, was excluded from the analysis due to failure of the qPCR reaction.

Our studies indicate that 7-d rosiglitazone treatment promotes both differentiation and browning of iWA cultures. It is common practice to induce adipocyte differentiation using an adipogenic cocktail, generally containing IBMX, dexamethasone, insulin, high glucose, and often 1 µM rosiglitazone, particularly in studies involving human adipocytes. However, the presence of rosiglitazone, and perhaps IBMX that increases cellular cAMP and thus mimics the effect of β-AR agonists, are confounding factors in any attempt to attribute browning capacity to agents being tested using cultures derived from the stromal vascular fraction (14). Several published studies have included rosiglitazone in mouse adipocyte cultures only for the first 2–4 days before removing rosiglitazone from the media for the remainder of the differentiation protocol, instead of including rosiglitazone for the entire differentiation period [e.g., Ref. (19, 21–23, 33)]. We therefore examined changes in gene expression in both BA and iWA cultures treated with rosiglitazone for the entire 7 days compared to the first 3 days only (Figure 5). In iWA cultures, 3-d rosiglitazone promoted increases in Adrb3 mRNA that were 50% of those seen after the full 7-d treatment. 1–3 day rosiglitazone did not significantly alter expression of UCP1, Fabp3, Fabp4, or Cpt1b, in contrast to the substantial increases seen in 7d-treated cultures. We also tested the effect of CL316243 (1 µM, 24 h) on cultures differentiated with rosiglitazone for 3 or 7 days. CL316243 increased the expression of Ucp1 in 1–3 day cultures, whereas it had no effect on control cultures, indicating that 3 days does provide a suitable window to test for browning capacity. After 7 days in the presence of rosiglitazone, CL316243 treatment for 24 h did increase UCP1 mRNA further (although not significant in this cohort of adipocytes due to high variation observed), and also seen in Figure 3, however, there was no increase in Fabp3 or Cpt1b expression associated with 24 h CL316243. Thus longer exposure to rosiglitazone essentially masks some of the effects of CL316243.

### Modulation of Gene Expression in BA and iWA Cultured From the SVF Compared to *In Vivo* Adipose Depots

Our gene expression data and previous functional studies (29) indicate that although BA and iWA cultures are derived from adipocyte precursors in the SVF, the mature adipocytes retain distinct properties according to their site of origin. It has been shown recently that *in vivo* treatment of mice with rosiglitazone induces a population of mature UCP1-positive inguinal adipocytes that have a distinct profile compared to populations induced by *in vivo* treatment with CL316243 (34). This differs from our findings based on adipocytes derived from the SVF, as in our cultures prior differentiation with rosiglitazone was required in order for cells to induce expression of the β_3_-AR and thereby become responsive to CL316243. This suggests that the mature adipocytes residing in iWAT depots differ from those derived from differentiation of the SVF. We compared the regulation of gene expression by rosiglitazone in our cultures with that demonstrated by transcriptome sequencing of RNA from BAT and iWAT subjected to sympathetic stimulation due to cold exposure of mice for 2 days (26). We also compared the expression of key adipocyte and thermogenic genes in our cultures with that in freshly isolated BAT and iWAT (Table 2). These tissues were isolated from mice subject to mild thermal stress by housing at 22°C, rather than at thermoneutrality (35, 36).

**Figure 6:**
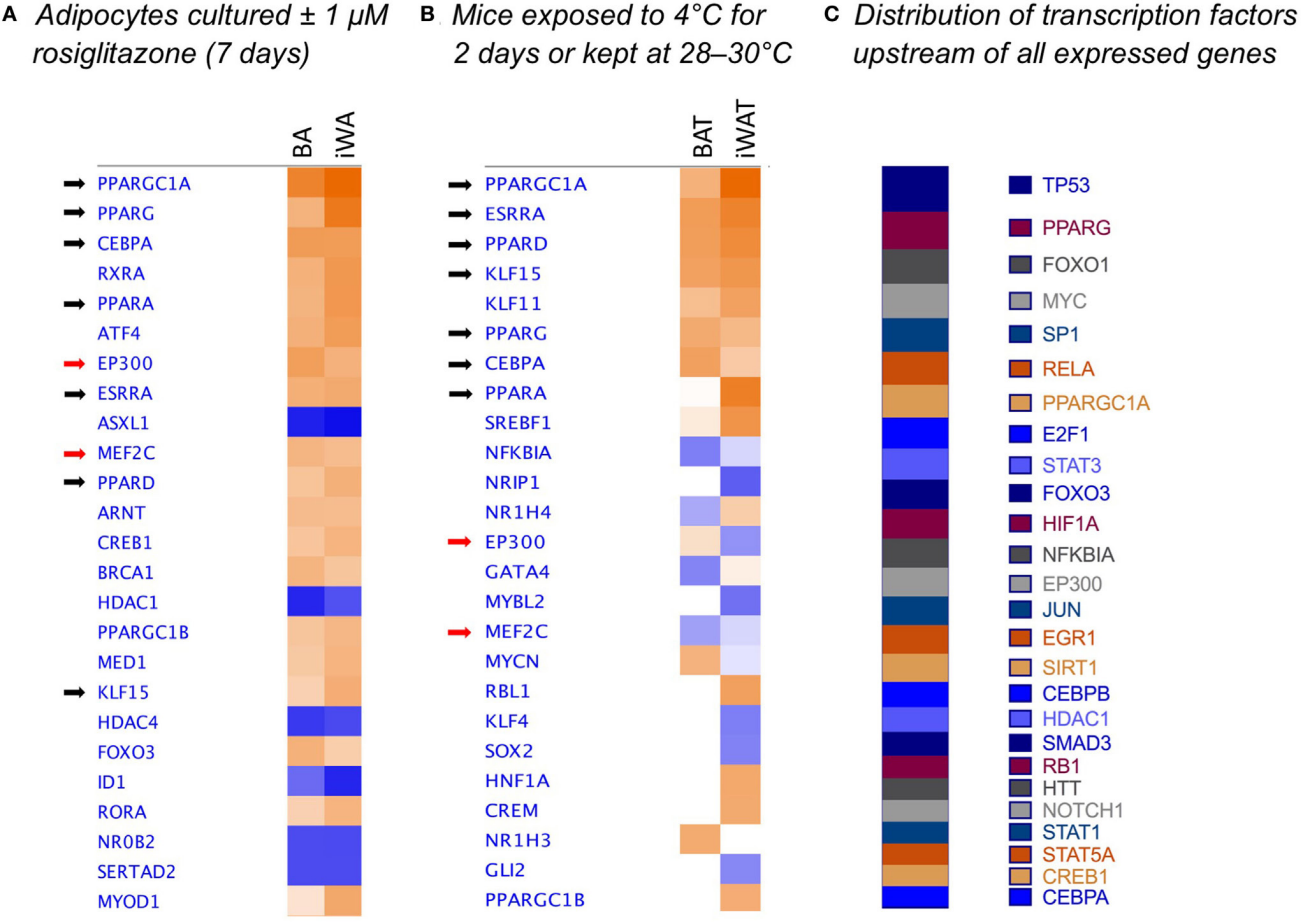
Comparison analysis [ingenuity pathway analysis (IPA)] of transcriptional regulators governing gene expression in rosiglitazone-treated adipocytes and 2-day cold-exposed mice. IPA software was used to determine upstream regulators consistent with observed patterns of target gene up- or down-regulation in adipocyte cultures treated with rosiglitazone relative to vehicle-treated control cultures **(A)**. Transcriptional regulators are ranked according to the sum of activation *z*-scores across brown adipocyte and inguinal white adipocytes (iWA) cultures, irrespective of positive or negative direction. *Z*-scores ≥|2| describe statistically significant matches between known regulators and observed patterns of up- and down-regulated genes, and also predict the activation state of each putative regulator (activated shown in orange, inhibited shown in blue) (25). As an example, the top transcriptional regulators in rosiglitazone-treated iWAs are PGC-1α (*z* = + 4.88, *P* value = 1.83E-46), PPARγ (*z* = + 4.35, *P* value = 5.89E-44), and PPARα (*z* = + 3.38, *P* value = 1.19E-32). These are predicted to be activated based on *z*-score, and are themselves upregulated in the treated cultures (83-, 3-, and 77-fold, respectively). *P* values (Fisher’s Exact Test) describe the significance of overlap between the observed differentially expressed genes and all genes associated with a particular upstream regulator in the Ingenuity database, curated from published literature. As an additional comparison, we re-analyzed literature-derived RNA sequencing data for adipose depots from cold-exposed mice (26) **(B)**. 8-week-old mice were housed at 28–30°C for 8 days, then one group maintained at thermoneutrality and a second group housed at 4°C for 2 days. Fold-change values were calculated for all genes expressed in iWAT and brown adipose tissue (BAT) of mice at 4°C compared to control mice at 28–30°C. These fold changes were analyzed using IPA and upstream regulators compared by *z*-score between BAT and iWAT. **(C)** Shows the predicted upstream regulators across the entire set of 171 genes found to be expressed in adipocyte cultures, ranked by log(*P*-value of overlap), demonstrating that the dataset is not biased toward the regulators predicted to govern differential expression in adipocyte cultures.

We used IPA to compare upstream regulators associated with all BA and iWA genes differentially expressed in the presence of rosiglitazone (twofold change in either direction, *P* < 0.05). As would be expected for cells treated with rosiglitazone, high-scoring upstream regulators across all cultures were closely associated with the PPARγ/PGC-1α network previously implicated in activating thermogenesis (Figure 6A). We also performed IPA upstream regulator analysis on the data from RNA sequencing of adipose depots from cold-exposed mice [(26), Figure 6B]. In BAT and iWAT, seven of the highest scoring upstream regulators from the *in vivo* study corresponded to those seen in our rosiglitazone-treated cultures. This highlights the previously characterized thermogenic pathway involving activation of PPARγ by endogenous fatty acids released due to sympathetic stimulation of lipolysis (37). When we compared expression of key adipocyte and thermogenic genes in control and rosiglitazone-treated cultures with that in native AT depots, many showed similar levels in rosiglitazone-treated cultures and in BAT or iWAT. There are, however, striking differences in levels of UCP1, Acot11, and Pck1 mRNA, which each display over 10-fold higher expression in native BAT and iWAT than in the corresponding rosiglitazone-treated cultures (Table 2; Figure 6).

## Discussion

The presence of active BAT in humans (38–41) has reignited research into ways to promote increased thermogenesis in humans as a strategy to combat obesity and its related disorders such as type 2 diabetes. This is in part due to the overwhelming evidence that activation of β_3_-ARs leading to increased UCP1 expression and function in BAT can reverse obesity, and prevent the development of insulin resistance and diabetes in rodents (42–45). However, translation of these responses observed in rodents has not led to effective treatments in humans (46, 47). This may in part be due to human tissue comprising adipocytes that are more akin to mouse beige or brite adipocytes rather than classical BAs, based on gene expression profiling (9, 48). Thus research into beige/brite adipocytes, and how they can be induced by different stimuli [reviewed in Ref. (14)] may offer greater therapeutic potential. It is critical to this research that model systems comprising cultured adipocytes respond in the same way to browning agents as endogenous cells *in vivo*.

We have characterized FVB/N mouse adipocytes induced by rosiglitazone in culture, utilizing a method for *in vitro* differentiation (8) that in control cultures lacks potential brite-inducing agents commonly used in adipocyte differentiation cocktails (such as triiodothyronine, IBMX, and rosiglitazone itself). We show that BA undergo differentiation in control cultures, but still display increased expression of UCP1 mRNA and protein in the presence of rosiglitazone. iWA cultures undergo both differentiation and browning due to inclusion of rosiglitazone. In BA cultures treated with rosiglitazone, 80% of cells become UCP1-positive, while this figure is 45% in iWA cultures (Figure 3B). These findings align with our observation that the key transcriptional regulators Prdm16 and PPARα are expressed at somewhat lower levels in iWA. Importantly, PPARγ and PGC-1α show comparable robust expression in rosiglitazone-treated BA and iWA (Figure 1).

### Cultured Adipocytes From iWAT Depots Require Priming by Rosiglitazone for Induction of UCP1 and Thermogenic Genes by the β^3^-AR Agonist CL316243

We showed previously that the majority of BA cells express β_3_-AR protein in control and rosiglitazone-treated cultures, whereas in iWA cultures the proportion of β_3_-AR-positive cells increase markedly in the presence of rosiglitazone (29), concomitant with increased levels of β_3_-AR mRNA (Figure 5). This in turn facilitates the response to β_3_-AR agonists (29). This may represent a special case, however, it is equally likely that receptors targeted by other agents are similarly induced during adipocyte differentiation and/or browning. In mice, the β_3_-AR is expressed in native BAT and iWAT at levels similar to those in rosiglitazone-treated cultures (Table 2), consistent with widespread findings that *in vivo* treatment with CL316243 can induce browning in iWAT depots (9, 35, 49). The utility of cultured adipocytes as a screening platform is clearly dependent on the presence of the target receptors.

Wu et al. (9) suggested that the inherent capacity for brite adipogenesis *in vivo* is independent of external factors, such as innervation, blood flow, oxygen supply, and nutrients. While differentiation of cultured adipocytes from the SVF does occur in a cell-autonomous manner, properties of the precursor cells appear to be specified by the environment from which they were derived, with a significant contribution from SNS innervation. *In vivo*, BAs possess a well-established ability to adapt to chronic β-AR activation; prolonged exposure to cold increases UCP1 expression in BAT (50, 51), which is inhibited by sympathetic denervation of BAT (37, 52). This upregulation in UCP1 occurs *via* β_3_-ARs and can be inhibited by β_3_-AR selective antagonists (53, 54). The requirement for intact sympathetic innervation has also been demonstrated in iWAT. For example, 3-week-old mice have high levels of UCP1 expression in iWAT, but this is markedly reduced by 8 weeks of age (35). At 8 weeks, mature adipocytes within iWAT re-express UCP1 in response to CL316243 stimulation, but this effect is abolished in mice undergoing prior surgical denervation of iWAT depots at 3 weeks. Thus sympathetic tone due to the mild cold-stress associated with housing mice at room temperature (55) maintains browning capacity in iWAT. We observed high UCP1 expression in native iWAT from animals housed at 22°C, reflecting this thermal stress (Table 2). Conversely, UCP1 mRNA is undetectable in the iWAT of mice housed at thermoneutrality [28–30°C (56)].

Importantly, reports of CL316243-induced browning have used mice housed at room temperature rather than at thermoneutrality (9, 35, 49). Previous studies have demonstrated an *in vitro* effect of CL316243 on UCP1 expression and function only in white adipocytes cultured in the presence of rosiglitazone or adipogenic cocktails (9, 49). Likewise, we found that while 24 h β_3_-AR stimulation (NE or CL316243) increased the proportion of UCP1-expressing cells in both control and treated BA cultures, neither agent alone induced significant browning of iWA cultures (Figure 3). These findings may recapitulate the requirement for intact SNS innervation of WAT seen *in vivo*, as sympathetic tone and consequent β-AR activation would be expected to drive lipolysis and subsequent activation of PPARγ by endogenous fatty acids. From our overall profiling of gene expression using the custom qPCR array, we examined predicted upstream regulators (IPA) based on genes differentially expressed following 7d rosiglitazone in cultured adipocytes, and these regulators were compared to those predicted in BAT and iWAT of mice subject to cold exposure for 2 days (26). In both cases, the top upstream regulators included PGC-1α, PPARγ, PPARα, PPARδ, CEBPα, ERRα (estrogen-related receptor α), and KLF15 (Figure 6), consistent with a common mechanism of induction that is dependent on PPARγ.

### Functional Demonstration of iWA Browning

The therapeutic potential of adipocyte browning is contingent upon the dissipation of energy through mitochondrial uncoupling becoming dominant over energy storage as triglycerides. We previously have shown that increased UCP1 function in brite adipocytes (defined as the percentage of OCR due to proton leak) is increased in brite adipocytes as compared to white adipocytes (29), which correlates with an increase in the expression of several genes involved in energy production (Figure 1). To assess whether combined treatment of iWA cells with rosiglitazone and a β-AR agonist (2 or 24 h treatment with either NE or CL316243) could further increase UCP1 function, we analyzed our OCR data, as described by Collins and colleagues (57), by defining the mitochondrial capacity that is not coupled to ATP synthesis. Rosiglitazone treatment of iWA induces a significant increase in this non-coupled spare capacity at the level of mitochondria, which is further increased in iWA also treated with either NE or CL316243 (Figure 4). These results indicate that the combination of both a browning agent (rosiglitazone) and a β_3_-AR agonist are required for maximal effects on mitochondrial function in brite adipocytes. This demonstrates that inguinal-derived brite adipocytes are geared toward mitochondrial uncoupling (and energy expenditure), consistent with a previous report that brite adipocytes are thermogenically competent, in that their mitochondria are functional and able to uncouple *via* UCP1 (58).

UCP1 is activated upon release of fatty acids generated following adrenergic stimulation of lipolysis, however, a recent study has also demonstrated that UCP1 activity is sensitized in the presence of ROS due to sulfenylation at Cys253 (59). *In vivo*, increased ROS due to deletion of Sod2 (encoding Mn-superoxide dismutase) leads to enhanced expression of key fatty acid oxidation genes in iWAT, and elevated mitochondrial oxygen consumption (60). Furthermore, UCP1 plays a central role in protecting adipocytes from mitochondrial dysfunction in the presence of high ROS and calcium overload (61). Thus, UCP1 is both activated by and protects against the effects of increased mitochondrial ROS. Our gene expression profiling and IPA indicates that rosiglitazone treatment may have dual protective actions in iWA, by increasing expression of UCP1 and at the same time inducing Sod1, Sod2, and other genes implicated in reduced ROS synthesis (Figure 2C).

### Culture Conditions That Promote Adipogenesis but Not Browning

Another issue raised by our study is whether it is possible to separate the effects of rosiglitazone on adipocyte differentiation from those on thermogenic programming in iWA. Certainly 7d rosiglitazone treatment promotes induction of UCP1, with further substantial increases following an additional 24 h in the presence of CL316243 (Figures 3 and 5). In contrast, Fabp3 and Cpt1b are increased by 7d rosiglitazone but there is no potentiation of expression by CL316243, indicating that responses to the β_3_-AR agonist are in part masked by prolonged rosiglitazone treatment. The magnitude of responses was smaller when rosiglitazone was included in the cultures only for the first 3 days of the 7-day culture period, however, this protocol did provide an improved window for observing the browning effect of 24 h CL316243 over and above rosiglitazone alone, with significant increases in expression of UCP1 (96-fold). The capacity to respond to CL316243 in these 3d cultures may be dictated solely by rosiglitazone-induced expression of the β_3_-AR, or it may reflect other aspects of the differentiation process. We cannot say at this time whether the 3d iWA most closely resemble the brite adipocytes seen *in vivo* (34), though profiling both 3d and 7d cultures would be worthwhile as it would facilitate a better understanding of the relationship between these adipocytes and those induced by PPARγ activation in mice.

## Conclusion

While there is a wealth of data on the gene expression of brite adipocytes (8, 9, 56, 62–66), these studies have generally aimed to identify differential genetic markers between brown, white, and brite adipocytes. We have instead sought to determine the similarities of brite adipocytes to conventional BAs in metabolic responses and profiles of thermogenic gene expression. We have demonstrated that the induction of brite adipocytes involves the upregulation of metabolic genes associated with thermogenesis, along with expression of β_3_-ARs, and this allows for adrenergically mediated activation of UCP1. Furthermore, brite adipocytes display the adaptive capacity observed in BAs, capable of responding to sustained adrenergic stimuli. Our findings indicate that cultured white adipocytes derived from regions such as iWAT with adequate sympathetic innervation respond efficiently to agonists, but only in combination with an additional priming stimulus such as rosiglitazone. This contrasts with *in vivo* iWAT depots in which chronic sympathetic tone is sufficient to maintain the capacity for brite activation. Our results emphasize the importance of activating both browning and thermogenic programs in cultured white adipocytes in order to reach maximum browning capacity. This understanding will translate into better model systems for the screening of beige or brite capacity to combat obesity and type 2 diabetes.

## Ethics Statement

All experiments were conducted with ethical permission from the Monash University Animal Ethics Committee, ethics approval numbers MIPS.2015.14 and VCP.2009.22, which complied with the National Health and Medical Research Council of Australia (NHMRC) guidelines for use of animals in scientific research.

## Author Contributions

JM designed and performed most of the experiments with assistance from MS and CN (confocal microscopy). RF, MP, and LC (qPCR), DH (adipocyte cultures, Seahorse studies), and BE (IPA analysis). JM, BE, and DH wrote the manuscript with help and suggestions from RS and TB. DH, BE, and TB conceived the research. All authors discussed the results and commented on the manuscript.

## Conflict of Interest Statement

TB owns stocks in the following pharmaceutical companies: Sigrid Therapeutics AB, Atrogi AB, and Glucox Biotechnology AB. DH owns stocks in Glucox Biotechnology AB. DH and RS are consultants for Atrogi AB.
